# Blockchain Enabled Anonymous Privacy-Preserving Authentication Scheme for Internet of Health Things

**DOI:** 10.3390/s23010240

**Published:** 2022-12-26

**Authors:** Arun Sekar Rajasekaran, Azees Maria, Maheswar Rajagopal, Josip Lorincz

**Affiliations:** 1 Department of ECE, KPR Institute of Engineering and Technology, Coimbatore 641407, India; 2 School of Computer Science and Engineering, VIT-AP University, Inavolu, Beside AP Secretariat, Amaravathi 522237, India; 3 Department of ECE, Centre for IoT and AI (CITI), KPR Institute of Engineering and Technology, Coimbatore 641407, India; 4 Faculty of Electrical Engineering, Mechanical Engineering and Naval Architecture (FESB), University of Split, 21000 Split, Croatia

**Keywords:** wireless, IoT, health, private key, attack, secure, privacy, sensor, network, cryptography

## Abstract

The Internet of Health Things (IoHT) has emerged as an attractive networking paradigm in wireless communications, integrated devices and embedded system technologies. In the IoHT, real-time health data are collected through smart healthcare sensors and, in recent years, the IoHT has started to have an important role in the Internet of Things technology. Although the IoHT provides comfort in health monitoring, it also imposes security challenges in maintaining patient data confidentiality and privacy. To overcome such security issues, in this paper, a novel blockchain-based privacy-preserving authentication scheme is proposed as an approach for achieving efficient authentication of the patient without the involvement of a trusted entity. Moreover, a secure handover authentication mechanism that ensures avoiding the patient re-authentication in multi-doctor communication scenarios and revoking the possible malicious misbehavior of medical professionals in the IoHT communication with the patient is developed. The performance of the proposed authentication and handover scheme is analyzed concerning the existing state-of-the-art authentication schemes. The results of the performance analyses reveal that the proposed authentication scheme is resistant to different types of security attacks. Moreover, the results of analyses show that the proposed authentication scheme outperforms similar state-of-the-art authentication schemes in terms of having lower computational, communication and storage costs. Therefore, the novel authentication and handover scheme has proven practical applicability and represents a valuable contribution to improving the security of communication in IoHT networks.

## 1. Introduction

Due to the growing and aging of the general population, healthcare has been confronted with a number of new issues. The Internet of Health Things (IoHT) is a key component in the Internet of Things (IoT) healthcare applications, where wireless transmission of sensing signals over air serves as a channel for data transmission between entities (patients and medical staff). The IoHT is an innovative solution that can serve the demands of both local and remote medical applications. In today’s context, the IoHT uses sophisticated sensors and wearable devices in combination with cloud computing, IoT and wireless networking to gather real-time biological data from the patient’s body. The IoHT, as part of a smart healthcare system [1,2], can provide excellent medical monitoring options for different categories of patients, particularly the elderly. The IoHT has been developed for healthcare systems using advanced information and communication technology. The IoHT offers a variety of monitoring services in the healthcare industry, allowing doctors to have a closer status of specific medical parameters of their patients [3,4,5,6,7]. The implementation of the IoHT technology is low-cost and uses non-invasive medical devices. Moreover, the IoHT is an important component of mobile health monitoring systems and dramatically improves healthcare quality and efficiency.

In monitoring the health conditions of elderly people or general people with some health problems remotely, the IoHT starts to play an important role. For example, in the IoHT networks, sensor nodes collect health information such as the patient’s pulse, heart rate, blood sugar and other symptoms of possible sickness. For the purpose of monitoring, diagnosis or treatment, such information is transferred to remote servers that are accessible to healthcare specialists via communication technology. Since the first introduction of the IoHT as a concept, there has been a major focus on increasing the security of data transfer, while reducing the IoHT communication and computation costs. Sensing devices in the IoHT transmit information about the human body at any time and from any location. Therefore, the reliable sending of sensing information of the individual is of vital importance. 

For monitoring health-related data from a remote distance, the information collected from the sensors should be transferred securely by means of a wireless medium. However, in the real-life implementation of the IoHT technology, the confidential data of the patients can be hacked by intruders and this imposes a serious security problem. To overcome such security challenges, in this paper a novel authentication scheme is proposed as an approach for achieving efficient authentication of the patient without the involvement of a trusted entity.

The IoHT network is generally made up of four parts: the transmitter, the receiver, the battery and the central processing server. Physiological sensors, environmental sensors and biokinetic sensors are types of sensors that are used to monitor real-time data related to human health and well-being. The main goal of the IoHT is to simplify and increase the speed, precision and reliability of sensor/actuator communication within, on or near a human body. The IoHT has the capability to communicate with the Internet as well as other wireless technologies such as ZigBee, wireless sensor networks (WSNs), Bluetooth, video surveillance and mobile cellular networks. There are two different types of sensors used in practical IoHT applications. The first is *in-body communication sensors* where the sensors or nodes for establishing IoHT communication are positioned inside the human body. The medical implant communication system is used for this purpose. The second application of sensors is *on-body communication sensors,* where interaction between wearable devices and the body occurs mostly through sensory components that are implanted in the human body [8,9,10].

The IoHT must include several required key features such as *trustability,* low *transmission latency, security, confidentiality, integrity and availability*. *Trustability* means that medical data of high precision is included in the data transmission chain by IoHT wearable devices or sensors, and the source of this data must be trustable. *Transmission latency* takes into account that some medical applications that deal with emergency data are not designed to sustain long response times. As a result, assured minimal transmission latency or real-time transmission is required. *Security* is related to the fact that the system should be capable of handling personal and sensitive data and data security and secrecy must be ensured. *Confidentiality* assumes that only authorized persons can have access to the data, and they must be validated by some authentication process before accessing it. Furthermore, data secrecy must be guaranteed in any phase of data processing, i.e., during the data transmission and storage phase. *Integrity* ensures that no unauthorized party should be allowed to alter sensing data or central processing device configurations. Furthermore, the data’s source should be reliable. *Availability* means that the information and sensing devices must always be available to authorized organizations and an unauthorized person(s) must not be able to interrupt communication or create a negative impact on the equipment. 

The IoHT technology is used in both medical and non-medical applications that support health monitoring. Medical applications are characterized by health monitoring devices that are dedicated to monitoring human medical parameters (heart rate, blood pressure, electrocardiogram (ECG), etc.). Examples of non-medical applications include navigation, time, distance, direction, surrounding temperature, etc., and that information may all be monitored using non-medical sensor devices such as sports sensors. Monitored information is through a concept known as telehealth care used to deliver healthcare over long distances by means of information and communication technology (ICT). Therefore, the IoHT as a technology is legal, affordable and easy to use. 

There are several advantages of wireless IoT networks over wired networks, including the elimination of lengthy wired communication lines and the threat of the entire system collapsing if parts of the network or specific node fails. Despite the various advantages of wireless IoT networks, the IoHT has some disadvantages such as limited storage capacity, susceptibility to the impact of noise or interference and continuous power supply issues. 

However, cloud computing arose as a solution to the IoHT technology’s limited storage capacity. Several networking concepts are commonly employed in the healthcare industry to deliver real-time patient monitoring and services. Although the medical expert may access a patient’s cloud-based data from any location on the planet, the patient’s sensitive data are transmitted over insecure cloud-based networks. Since only legitimate users have access to their data and services, there is a need for a strong user authentication system. Due to the fact that the physiological parameters of patients are extremely sensitive in terms of privacy, secure communication in the IoHT networks is of great importance. Additionally, security risks arise as a result of the open nature of cloud computing and wireless connectivity. Secure user authentication is thus required because the patient’s data is sent over insecure Internet networks [11,12]. Therefore, data security methods are established using some components such as transmission over trusted gateway devices or other highly reliable components and through introducing different authentication schemes for securing the IoHT data transmission. The trusted device may be a smartphone, a computer or an IoT device that is connected to the concentrator device using some of the wireless network types which can include proprietary IoT networks (LoRa, Sigfox, NB-IoT, etc.), the 3rd generation (3G)/ 4th generation (4G)/ 5th generation (5G) cellular networks, wireless local area network (WLAN) or satellite communication. 

Moreover, several authentication techniques for the IoHT have been proposed in recent years to improve the security of the IoHT data transmission through securely encrypting patients’ confidential medical data and transmitting it to medical advisors. To contribute to these attempts, this work proposes a lightweight blockchain-based authentication scheme that offers protection against a variety of security risks. More specifically, the main goal of this work is to ensure the efficient transfer of the confidential information of the patient to medical professionals (doctors) and to send the confidential medical prescription from the doctor to the patient through the development of a novel authentication scheme. Furthermore, secure handover authentication is suggested to avoid the re-authentication of patients when they move from one location to another.

Therefore, the main contributions of this work are:

1. Development of an authentication scheme that guarantees efficient anonymous authentication for patients and medical staff, where confidential biological information is accessed only by authenticated doctors or patients.
2. Development of an authentication scheme that guarantees integrity and data confidentiality of both the confidential biological information and medical prescription of patients and doctors from attackers.
3. Development of an authentication scheme that guarantees an efficient revoking mechanism for malicious misbehaving of medical staff in the IoHT wireless body area network.
4. Development of an efficient authentication handover that enables avoiding re-authentication of the patients when new doctors start their health monitoring process.

The rest of the manuscript is structured as follows. Section 2 describes some of the prevalent authentication schemes in IoT networks. Basic preliminaries of the methods used for the development of the proposed authentication scheme are introduced in Section 3. The operating methodology of the proposed authentication scheme is described in Section 4. Security analysis is explained in Section 5. Performance study and comparison with other prominent IoHT authentication schemes in terms of computational, communication and storage costs are analyzed in Section 6. Finally, Section 7 concludes the manuscript. 

## 2. Related Works

Many authors have concerted on providing an efficient, secure, anonymous protocol to provide security among IoHT users. Identification (ID)-based public key was suggested by Wang et al. in [13] where the private secret key of the user is computed by the key generator based on the identity of the user. This scheme encountered key escrow problems and vulnerability to several security assaults. Zhao et al. in [14] suggested an elliptic curve-based authentication scheme for IoHT users. However, this scheme proves to be vulnerable in securing the privacy and anonymity of IoHT users. To compensate for these drawbacks, Omala et al. in [15] suggested an authentication scheme based on remote protocol. The anonymity of the end users and security against impersonation attack is achieved in this work. Several authentication schemes based on authentication and key agreement protocol are suggested in the works [16,17,18]. These works mainly focus on the unlinkability between the end users and forward secrecy having the main drawback in the reply attack. 

Song in [19] has developed a novel smart card-based password authentication system. Based on the upgraded smart card authentication approach, this scheme demonstrates that it is impossible for an adversary to retrieve the information. Additionally, it is challenging for an attacker to masquerade as a genuine authenticated user. In this study, the symmetric approach is used to encrypt both the server’s secret key and the user’s actual identity. Li et al. in [20] offer a solution for forward secrecy and password detection. The biggest disadvantage is that the user cannot change the password without the trusted authority’s consent. The vulnerability of the scheme proposed in [19] was demonstrated by Chen et al. in [21], and according to demonstrated results, if the smart card is missing, it results in a password-predicting attack. Additionally, although mutual authentication between the end users is provided in [21], password detection during the login step is the primary downside. A unique RSA-based authentication technique was proposed by Sutrala et al. IN [22] to protect end users’ anonymity. This work is resistant to a variety of attacks, including impersonation attacks, password-guessing attacks and reply attacks. However, as compared to other methods of a similar nature, the proposed scheme has a relatively high communication cost. Tanmoy et al. in [23] proposed an effective elliptic curve cryptography (ECC)-based smart card authentication method. In this research, user anonymity is maintained. However, this technique is vulnerable to attacks including password guessing. An authentication system based on a multi-cloud server environment was proposed by Saru et al. in [24]. This work uses biometric authentication as its foundation. Furthermore, this approach makes advantage of biohashing. According to the user’s convenience, the password can be changed at any time. However, this effort does not specifically address the security issues of cloud servers. Feng et al. in [25] proposed a biometrics-based authentication method for multi-cloud server environments, which addressed the shortcomings of Saru et al. [24]. However, this technique is vulnerable to known session key attacks.

A new and enhanced smartcard-based authentication system was developed by Islam [26]. His proposed work fixes the problems authentication scheme proposed by Li et al. in [20]. However, involving the proposed procedure has a significant communication and computational cost. An improved authentication mechanism with increased security was proposed by Kaul and Awasthi in [27]. This work has proven that the proposed mechanism is safe for several potential well-known attacks, including impersonation attacks, bogus message attacks and session key assaults. However, the execution of this strategy comes at a considerable computational cost. Additionally, this technique is vulnerable to password-guessing attacks. An effective RSA cryptosystem was proposed by Amin et al. in [28] for distant user authentication. The proposed system is resistant to both active and passive attacks. However, this work is vulnerable to impersonation and password-guessing attacks. An identity-based authenticated approach was proposed by Luo et al. in [29]. Mutual authentication using a smart card is carried out in the proposed approach. However, the technique has a significant computational cost and is vulnerable to man-in-the-middle and session key attacks.

In a multi-server context, Ali and Pal in [30] have recommended a three-factor authentication system to improve security. However, there is a significant communication overhead in this approach. The technique is resistant to a variety of attacks, including biometric and session key intrusions; however, it is vulnerable to known session key attacks and lacks secrecy. A strong biometric-based authentication method was put forth by Qi and Chen in [31]. In the case of this method, security is aided by mutual authentication between the entities. The method offers full confidentiality and is resistant to denial-of-service attacks. However, the method can be used in a single-client scenario. When the same protocol is used in a multi-server context, there is a significant increase in computational and communication overhead. Additionally, this approach is vulnerable to password guessing and impersonation assaults. For telecare medicine, Sharif et al. in [32] proposed a mutual authentication system based on ECC. In this work, a novel patient authentication system and key agreement protocol are devised to provide access to the medical server. The recommended strategy defends against both aggressive and passive attacks. However, the recommended technique has a high computational cost for both server and mobile device authentication.

To overcome different security threats Xu et al. in [33] suggested a novel authentication scheme with privacy preservation. This scheme can withstand against several possible security threats such as impersonation and reply assaults. However, forward secrecy and confidentiality of the transferred information are not achieved in this work. Xiong et al. in [34] focus on the certificateless signature and encryption scheme with an efficient revoking mechanism. The computational burden due to the key updation is reviewed in this work. Though an efficient revoking mechanism is adopted, this work lacks a conditional tracking mechanism. Zhou et al. in [35] propose a certificateless key scheme that is computed based on the private key of the key generator and user. Saeed et al. in [36] focus on the certificateless online/offline signature scheme for IoHT users. Remote authentication protocol based on IoT is used in this work. The authors claim the scheme is secure against several attacks, but its vulnerability to forgery attacks is proved by Liao et al. in [37]. Ji et al. in [38] suggested a work based on big data analysis of body area networks. Conditional transmission privacy, mutual and batch authentication and un-linkability are achieved in this work. However, this work does not support handover authentication and removal of misbehaved doctors/patients. Vijayakumar et al. in [39] mainly focus on the location privacy of the end users without addressing the transfer authentication and revoking mechanisms. 

Son et al. in [40] discuss the telecare medicine system. A ciphertext encryption policy is used in this work for access control of medical data. Data integrity is ensured using blockchain technology. However, there is no revoking mechanism to remove the misbehaving medical professionals or patients in the network. Zhang et al. in [41] mainly focus on the conditional privacy of the end users. The true identity of the patient is hidden in the cloud-based medical network. Moreover, the blockchain-based protocol is used for storing the data which avoids tampering with data. 

Peng et al. in [42] suggested a certificateless signature scheme to overcome the resource-constraint nature of the sensor unit. The size of the signature used in this work is similar to the related prevailing works. However, this work fails to revoke the malicious end users from the medical network. Lara et al. in [43] proposed a two-party authentication scheme based on elliptic cryptography. Though this method uses the lightweight authentication protocol, there is no efficient handover and revocation mechanism in this work. Kumar et al. in [44] focus on cloud-assisted technology to improve storage capacity. Due to the limited storage capacity of the mobile unit controller, a large volume of collected data cannot be stored and analyzed. To overcome this issue, gathered data is stored in the cloud storage; however, the security of maintaining the information in the cloud is questionable. Moreover, the computational cost of this work is comparatively high. 

Although presented related works show improvements in terms of the development of authentication schemes, the main research gap is the lack of authentication schemes that offer a combination of secure re-authentication of the patients (adopting a handover mechanism) and revocation of the misbehaving doctors. This paper tends to fill this gap with the introduction of a novel authentication scheme dedicated to improving authentication efficiency and reducing computing costs. In this work, this research gap tends to be fulfilled by proposing a solution that is based on the blockchain concept. Confidential information is stored in the blockchain and only authenticated IoHT users can access this data. If any intruder tries to hack the block, this will have an impact on the subsequent blocks affecting the entire blockchain network. When the patient moves from one location to another location without the involvement of a trusted authority, the new doctor takes the data of the patient from the blockchain. As a result, there is no re-authentication of the patient, which results in a reduction in the authentication time.

Moreover, a revocation mechanism is adopted in a way that when the misbehaving doctors (attackers) are identified by the trusted authority, their fake identities are loaded into the blockchain list. Hence, the misbehaving doctor will not be allowed to proceed further in the IoHT network. The results of the performance comparison in terms of computational, communication and storage overhead of the proposed authentication scheme are compared with other known state-of-the-art authentication schemes. 

## 3. Development Methods of the Proposed Authentication Scheme

The methods on which the development of the proposed authentication scheme is based will be presented in this section and they include elliptic curve cryptography, bilinear pairing and blockchain in the IoHT network. Moreover, in this section, the analyzed system model will be presented.

### 3.1. Elliptic Curve Cryptography

The proposed authentication scheme exploits the concept of ECC. It is the concept of realization of public-key cryptography using the algebraic structure of elliptic curves over finite fields. Let us take an elliptic curve over a finite field demarcated by $E\left(i,j\right):p^{2}=r^{3}+ir+j\;mod\;q$, which gratifies the condition $4i^{3}+27j^{2}\neq 0$ and where $i,j\in Z_{q}^{*}$ under the group $G=\left\{\left(r,p\right):r,p\in Z_{q}^{*},\left(r,p\right)\in E\right\}\cup^{\;}\left\{⊚\right\}$. Here, ⊚ signifies the identity value under the additive group. Moreover, the scalar multiplication in ECC is denoted as nA=A+A+A+A+⋯A, where *n* denotes the private key value. The scalar point addition is denoted as $A+B=\left(r_{3,}p_{3}\right)$ such that $A=\left(r_{1},p_{1}\right)\in G$,$B=\left(r_{2},p_{2}\right)\in G$, where the values of $r_{3}$ and $p_{3}\;$ are calculated as follows:

(1) $$r_{3}=\lambda^{2}-r_{1}-r_{2}\;mod\;q$$

(2) $$p_{3}=\left(\lambda \left(r_{1}-r_{3}\right)-p_{1}\right)\;mod\;q$$

and constant λ equals:

(3) $$\lambda =\{\begin{array}{c} \frac{p_{2}-p_{1\;}}{r_{2}-r_{1}}mod\;q\;if\;A\neq B\\ \frac{3r_{1}^{2}+i}{2p_{1}}mod\;q\;if\;A=B \end{array}$$

### 3.2. Bilinear Pairing

Another relevant concept for executing the proposed authentication scheme is commonly used to construct and analyzed cryptographic systems. It is based on pairing between elements of two cryptographic groups ($G_{1},\;G_{2}$) to a third group with a mapping $:G_{1}*G_{2}\to G_{T}$. Let us consider $G_{1}$ and $G_{2}$ as the multiplicative cryptographic groups of prime order q. Let $Z_{q}^{*}$ be the multiplicative group of the finite field $F_{p}$ and the $e:G_{1}*G_{2}\to G_{T}$ be a bilinear map which gratifies the succeeding properties. Next properties can be achieved:

(1) Bilinearity: for any $A,B,C\;\in G_{1}$, e(A,B+C)=e(A,B)e(A,C) and e(A+B,C)=e(A,C)e(B,C).
(2) Non-degeneracy: for non-identify points $P,Q\in G_{1}$, $e\left(P,Q\right)\neq 1_{G_{T}}$, where $1_{G_{T}}$ is the identity point of $G_{T}$.
(3) Computability: for any two points $P,Q\in G_{1}$, there exists a polynomial time algorithm to determine the value of e(P,Q).

### 3.3. Blockchain Technology

The developed authentication scheme utilizes the concept of blockchain technology. In blockchain technology, information is stored in the form of blocks that are linked together in a secure way. Any modification of data in the block will affect the subsequent blocks. Thus, the data loaded in the blocks are immutable [45,46,47,48]. In our work, confidential information is stored in the blockchain and only the authenticated IoHT users can access the data. If any intruder tries to hack the block, it will affect the subsequent blocks affecting the entire blockchain network. The medical experts are responsible for providing the medical prescription to the patient. There may be a possibility that the medical expert/doctor can be corrupted, and as a consequence, send fake data regarding the patient to the subsequent doctor in the network. This will degrade the performance of the IoHT. 

In the proposed authentication scheme, the introduction of blockchain technology alleviates these problems. As an outcome, blockchain-integrated IoHT empowers authenticity and integrity, without the involvement of a trusted entity, thus decreasing the computational overhead. 

### 3.4. System Model 

**Figure 1:**
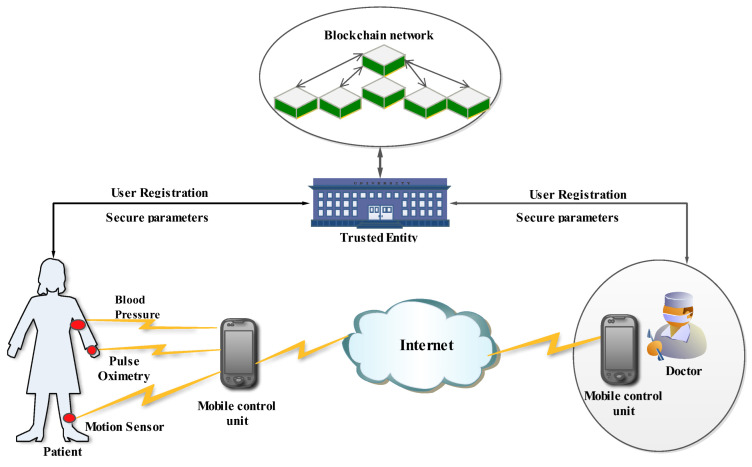
Proposed IoHT architecture.

The system model used for analyses is composed of three entities. Figure 1 illustrates the IoHT architecture of the analyzed system model. The three major entities of the analyzed system model are the trusted entity, the mobile control unit and the end users. 

The trusted entity (TE) is a completely trusted authority, and it cannot be compromised by anyone. It is responsible for generating the system parameters and providing the authentication parameters to the authenticated users in the IoHT network (Figure 1). Initially, both the patient and medical experts should register in the TE by providing their required original credentials. After their successful registration, the required parameters are given to the authenticated users by the TE. Moreover, the TE is responsible for adding malicious doctors to the blocklist. As a result, the malicious doctors are revoked from the IoHT and their further communication in the network is avoided.

The mobile control unit (MCU) is provided to both authenticated end users in the IoHT network (Figure 1). The MCU has a high capability of performing the computation, data storage and generation of system parameters. There are two different types of MCUs provided to the patient and the medical staff (doctor(s)). The MCU provided to the medical staff has the capability of encrypting the medical prescription and sending it to the patient’s device. Moreover, it will be responsible for collecting biological confidential information from the patient’s MCU. The patient’s MCU is embedded with a controller that is capable of collecting sensitive data from the sensors attached inside or on the body of the patient. This collected sensitive data are encrypted and sent back to the medical staff MCU (Figure 1). The patient MCU has an in-build analog to digital converter (ADC) for converting the analog sensor data collected from the sensors into digital signals and processing it in the MCU of the patient. 

The end users in the IoHT network are medical staff (professionals)/experts and patients (Figure 1). To become an authorized user in the network, they should be registered in the network. Only after their successful registration, they become authenticated users. Only the authenticated users are provided with a unique MCU by the TE. With the help of the MCU, the end users communicate in the network and transfer the required information between them (Figure 1). 

### 3.5. Security Objectives

The analyzed IoHT system model presented in Figure 1 can be susceptible to different security threats. The main security objectives can be categorized into the following five types:

- *Ensuring message integrity and authentication:* the confidential biological sensitive data of the patient or the medical prescription of the doctor should be integrity preserved. The possibility of forging or modifying the information by the intruder should be eliminated.
- *Ensuring nonrepudiation:* only authenticated end users are allowed to participate in the data transfer over the IoHT network. There must be no possibility for the registered users to deny the message transmission once it is sent.
- *Ensuring anonymity and privacy:* the real identity of the end users should be preserved during the transfer of confidential data. Moreover, the private confidential information of the end users should be preserved.
- *Ensuring unlinkability:* there should not be any correlation between the subsequent information sent between the end users.
- *Ensuring revocation and traceability:* if any mishap occurs in the network and the end user is trying to send fake information, the real identity of the end user should be traced immediately and revoked from the IoHT network.

## 4. Description of the Proposed Authentication Scheme

For an efficient transfer of confidential biological information between the patients and doctors in the current scenario, a blockchain-based integrity preservation scheme is proposed in this work. This scheme also achieves anonymous authentication between the end users. The notations and their description used in the further analyses are shown in Nomenclature.

The important steps that are carried out in the proposed scheme include initialization of the system, registration of both patients and doctors with a trustworthy network, Anonymous authentication, handover authentication, preservation of integrity and revocation. The entire flow diagram of the proposed authentication scheme is shown in Algorithm 1 and Algorithm 2, respectively. Algorithm 1 shows the phases related to the registration, the key generation and authentication of the patient, while Algorithm 2 shows the authentication of the doctor and handover authentication phases.

**Algorithm 1:** Flow diagram of registration, key generation and authentication of patient.
** part 1: the anonymous authentication of patient**

** *Initialization:***

* 1. Elliptic curve of finite field:*

$y^{2}=x^{3}+ax+b\;mod\;q$


* 2. Points on the curve:*

X


* 3. Random numbers*

$a,b\;\in Z_{q}^{*}$


* 4. Public parameter of TE:*

α=aX


* 5. Authentication parameter of TE:*

β=bX


* 6. Hash function generation:*

$H:\left\{0,1\right\}\to Z_{q}^{*}$

* 7. Public parameters:*(α, β,$H,X,e\left(X,X\right),q)$
** *Patient’s registration:***

* 8. TE chooses*

$\rho_{i},k\in Z_{q}^{*}$


*calculate*

$VID_{p_{i}}=\rho_{i}\left(a+b\right)$


*calculate*

$FID_{p_{i}}\in Z_{q}^{*}$

 9. $\left(\rho_{i},VID_{p_{i}},FID_{p_{i}},x_{1},x_{3}\right)$ 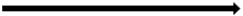

*Patient*
 10. $\left(FID_{p_{i}},Z\right)$
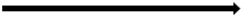

*Blockchain where*
$Z=e{\left(X,X\right)}^{\rho_{i}}$

** *Doctor’s registration:***
 11. TE
*chooses*
$c_{i},x\in Z_{q}^{*}$

*calculate*

$VID_{di}=\left(\frac{1}{a+b}\right)X$

*calculate*$FID_{di}$ $\in Z_{q}^{*}$ 12. $\left(VID_{di},FID_{di},x,y_{3},y_{5},y_{6},y_{7}\right)\;$
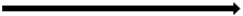
* Doctor*
** *Patient’s key generation:***

* 13. secret key is*

$Sk_{p_{i}}=x_{3}+H(x_{1}||FID_{p_{i}})\rho_{i}$


* 14. public validation key is*

$Pk_{p_{i}}=Sk_{p_{i}}.X$


** *Doctor’s key generation:***
 *15.*
$secret\;key\;is\;Sk_{d_{i}}=y_{5}+H(y_{1}||FID_{d_{i}})x$
 *16. public validation key is*
$Pk_{d_{i}}=Sk_{d_{i}}.X$

** *Anonymous authentication of Patient:***

***Patient***

***Doctor***
 17. $\rho_{i}X$  
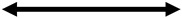


$FID_{di}X$

 18. $FID_{di}X$


$\rho_{i}X$

 19. $f=FID_{di}X.\;\rho_{i}$


$f=\rho_{i}X.FID_{di}$

 20. $f_{1}=VID_{p_{i}}⨁H\left(f\right)$
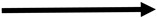


$VID_{p_{i}}=f_{1}⨁H\left(f\right)$

 21.
*Verifies*

$e\left(VID_{p_{i}}X,VID_{d_{i}}\right)=Z$

 22.

$AA=\left(\;FID_{p_{i}},\;FID_{d_{i}},H\left(\;FID_{p_{i}},\;FID_{d_{i}}\right)\right)$

 23_._
$FID_{d_{i}}=VID_{p_{i}}⨁f_{2}$
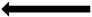


$f_{2}=VID_{p_{i}}⨁FID_{d_{i}}$



**Algorithm 2:** Anonymous authentication of doctor and handover authentication.
** part 2: the anonymous authentication of the doctor**


***Doctor***

***Patient***
1.Reception of parameters: $VID_{di},FID_{di},c_{i},x,b,y_{3},y_{5},y_{6},y_{7}$

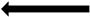

2.

$l_{1}=FID_{d_{i}}⨁y_{7}$


3.

$l_{2}=VID_{p_{i}}⨁y_{6}$


4.

$l_{1},\;l_{2},\;c_{i}$



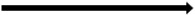


5.


$y_{6}=VID_{p_{i}}⨁l_{2}$

6.


$y_{7}=FID_{d_{i}}⨁l_{1}$

7.

***Verifies***

$y_{6}X=\left(y_{1}+y_{6}\beta \right)$



***Transfer of biotic information from the patient to the doctor***


***Patient***

***Doctor***
8.$t_{1},t_{2}$ $\in Z_{q}^{*}$
9.

$u_{1}=t_{1}\left(Pk_{p_{i}}+H\left(\omega \right)X\right)$


10.

$v_{1}=H(g^{t_{1}}||Pk_{d_{i}})$


11.

$m_{1}=t_{2}X$


12.

$m_{2}={\left[Sk_{p_{i}}+H\left(\omega \right)\right]}^{-1}X-t_{2}Pk_{d_{i}}$


13.$\left(u_{1},v_{1}\right)$,$\;m_{1}$ and $m_{2}$

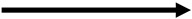

14.

***Verifies***

$\;e\left(u_{1},Sk_{d_{i}}\;m_{1}+m_{2}\right)=g^{t_{1}}$



***Transfer of medical prescription from the doctor to patient***


***Doctor***

***Patient***
15.$t_{3},t_{4}$ $\in Z_{q}^{*}$
16.

$u_{2}=t_{3}\left(Pk_{d_{i}}+H\left(\omega^{\prime }\right)X\right)$


17.

$v_{2}=H(g^{t_{3}}||Pk_{p_{i}})$


18.

$m_{3}=t_{4}X$


19.

$m_{4}={\left[Sk_{d_{i}}+H\left(\omega^{\prime }\right)\right]}^{-1}X-t_{4}Pk_{p_{i}}$


20.$\left(u_{2},v_{2}\right)$, $m_{3}$ and $m_{4}$

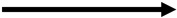

21.

***Verifies***

$\;e\left(u_{2},Sk_{p_{i}}\;m_{3}+m_{4}\right)=g^{t_{3}}$



***Handover authentication***


***Current doctor***

***Next doctor(s)***
22.

$d_{1},\;d_{2},d_{3}\in Z_{q}^{*}$


23.$D_{1}=d_{1}\alpha$, $D_{2}=d_{2}\alpha$, $D_{3}=D_{1}+D_{2}$, $D_{4}=d_{3}\alpha$,$\;D_{5}=D_{3}+D_{4}$
24.

$\;Q_{i}=\partial_{i}\left(\;d_{1}+d_{2}+d_{3}\right)mod\;q$

*where*

$\partial_{i}=H\left(\omega \times D_{4}\right)$


25.

$\;\sigma =\left(D_{4},\omega \right)$


26.

$\left(Q_{i},TS,\omega ,\sigma ,D_{3},FID_{pi}\right)$


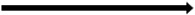



$\partial_{i}=H\left(\omega \times D_{4}\right)$

27.

***Verifies***

$Q_{i}\alpha =\partial_{i}D_{5}$



### 4.1. System Initialization

In the phase of system initialization, an elliptic curve of finite field $y^{2}=\left(x^{3}+ax+b\right)mod\;q$ is chosen by the TE, where q is the largest prime value (line 1 in Algorithm 1). Let the X denote the point on the finite elliptic curve (line 2 in Algorithm 1). In the next phase, the TN chooses $a,b\in Z_{q}^{*}$ as the random numbers (line 3 in Algorithm 1). Let the $Z_{q}^{*}$ be the multiplicative group of size q. Moreover, the public parameter and the authentication parameter are calculated as α=aX and β=b (lines 4 and 5 in Algorithm 1). The system initialization phase ends with the TE publishing the parameters (α, β,H,X,e(X,X),q) to all the patients and doctors who joined the network (line 7 in Algorithm 1). Here, the hash function is given by H:{0, 1}* and e(X,X)=g (line 6 in Algorithm 1). The hash function is used for ensuring data protection of an individual’s privacy rights in the blockchain system. 

### 4.2. Patient’s Registration

The next phase of the developed authentication algorithm is the patient registration phase (Algorithm 1). Initially, the patients should be registered with the TE. Moreover, the patients should provide their confidential credentials such as an identification card, mobile number, address, etc., to the TE in an offline way. Once the credentials submitted by the patients are verified, TE chooses a random number $\rho_{i},k\in$ and calculates the validation ID and fake ID for each and every patient ($p_{i}$) as $VID_{p_{i}}$ and $FID_{p_{i}}$, where $VID_{p_{i}}=\rho_{i}\left(a+b\right)$ and $FID_{p_{i}}\in Z_{q}^{*}$ (line 8 in Algorithm 1). 

To communicate with everyone, a fake identity is used. Only the fake identity is exposed to other entities during data transfer. Moreover, in the TE, dummy identities are mapped to the true identities. Even if the fake identities are captured, they provide zero information about the true identities. Thus, the authorized user can anonymously authenticate the specific user and maintain privacy. The TE computes the following parameters $x_{1}=R+\rho_{i}X$, $x_{2}=H(x_{1}||FID_{p_{i}})$ and $x_{3}=a+b+x_{2}k$ (line 9 in Algorithm 1). Finally, the TE securely provides $\left(\rho_{i},VID_{p_{i}},FID_{p_{i}},x_{1},x_{3}\right)$ to the patients. Moreover, the TE stores $\left(FID_{p_{i}},Z\right)$ in the blockchain network, where $Z=e{\left(X,X\right)}^{\rho_{i}}$ (line 10 in Algorithm 1).

### 4.3. Doctor’s Registration

Similar to the patient’s registration, in the next phase of the proposed algorithm, it is mandatory for the doctors to register with the TE by giving the required credentials. The validation ID for each doctor is calculated as $VID_{di}=\left(\frac{1}{a+b}\right)X$ and the fake identity for every doctor is computed as $FID_{di}$ $\in Z_{q}^{*}$ by the TN (lines 11 in Algorithm 1). Moreover, the TN chooses two random numbers $c_{i},x\in Z_{q}^{*}$ and calculates the following parameters $y_{1}=c_{i}X$, $y_{2}=xX$, $y_{3}=y_{1}+y_{2}$, $y_{4}=H(y_{3}||FID_{di})$, $y_{5}=a+b+y_{4}c_{i}$, $y_{6}=H\left(VID_{di}\times \alpha \right)$ and $y_{7}=\left(c_{i}+y_{6}b\right)mod\;q$. Then, the TE provides the $\left(VID_{di},FID_{di},c_{i},x,b,\;y_{3},y_{5},y_{6},y_{7}\right)$ to the corresponding doctors (line 12 in Algorithm 1).

### 4.4. Patient’s Key Generation

After the doctor’s registration phase, the next algorithm phase that is executed is the patient’s key generation phase. In this phase, the secret key and public key are generated by the authenticated patient device based on the received values of $\left(\rho_{i},VID_{p_{i}},FID_{p_{i}},x_{1},x_{3}\right)$. The secret key is computed as $Sk_{p_{i}}=x_{3}+H(x_{1}||FID_{p_{i}})\rho_{i}$ (line 13 in Algorithm 1) and the public validation key is calculated as $Pk_{p_{i}}=Sk_{p_{i}}.X=\alpha +\beta +H(x_{1}||FID_{p_{i}})x_{1}$ (line 14 in Algorithm 1). Finally, the key pair is maintained as $\left(Sk_{p_{i}},\;Pk_{p_{i}}\right).$ Here, the public verification key is generated internally from the public and authentication parameters of TE.

**Proof of validation.**  $Pk_{p_{i}}=Sk_{p_{i}}.X\;=[x_{3}+H(x_{1}||FID_{p_{i}})\rho_{i}]X\;=x_{3}X+H(x_{1}||FID_{p_{i}})\rho_{i}X\;=x_{3}X+H(x_{1}||FID_{p_{i}})\rho_{i}X\;=\left(a+b+x_{2}k\right)X+H(x_{1}||FID_{p_{i}})\rho_{i}X\;=(aX+bX+(H(x_{1}||FID_{p_{i}}))kX+H(x_{1}||FID_{p_{i}})\rho_{i}X\;=(aX+bX+H(x_{1}||FID_{p_{i}})\left[kX+\rho_{i}X\right]\;=\alpha +\beta +H(x_{1}||FID_{p_{i}})\left[kX+\rho_{i}X\right]\;=\alpha +\beta +H(x_{1}||FID_{p_{i}})\left[kX+R\right]\;=\alpha +\beta +H(x_{1}||FID_{p_{i}})x_{1}$ □

### 4.5. Doctor’s Key Generation

Similar to the previous phase related to the patient’s key generation, in this phase, the secret key and public key are generated by the authenticated doctor’s device based on the received values from the TE. The secret key is computed as $Sk_{d_{i}}=y_{5}+H(y_{1}||FID_{d_{i}})x$ (line 15 in Algorithm 1) and the public validation key for the doctor is calculated as $Pk_{d_{i}}=Sk_{d_{i}}.X=\alpha +\beta +H(y_{3}||FID_{d_{i}})y_{3}$ (line 16 in Algorithm 1). Finally, the key pair is maintained as $\left(Sk_{d_{i}},\;Pk_{d_{i}}\right).$ Here, the public verification key is generated internally from the public and authentication parameters of TE.

**Proof of validation.**  $Pk_{d_{i}}=Sk_{d_{i}}.X\;=[y_{5}+H(y_{3}||FID_{d_{i}})x]X\;=y_{5}X+H(x_{1}||FID_{p_{i}})xX\;=\left(a+b+y_{4}c_{i}\right)X+H(y_{3}||FID_{d_{i}})xX\;=(aX+bX+(H(y_{3}||FID_{d_{i}}))c_{i}X+H(x_{3}||FID_{d_{i}})xX\;=(aX+bX+H(y_{3}||FID_{d_{i}})\left[c_{i}X+xX\right]\;=\alpha +\beta +H(y_{3}||FID_{d_{i}})\left[c_{i}X+xX\right]\;=\alpha +\beta +H(y_{3}||FID_{d_{i}})\left[y_{1}+y_{2}\right]\;=\alpha +\beta +H(y_{3}||FID_{d_{i}})y_{3}$ □

### 4.6. Patient’s Anonymous Authentication

The next phase of the proposed algorithm is dedicated to the patient’s anonymous authentication. The process of validating the credentials of patients and doctors in order to ensure security is known as authentication. The anonymous authentication process authenticates doctors and patients without disclosing their true identities. As a result, anonymous authentication protects end users’ privacy. In order to communicate with patients and other doctors, the MCU of the doctors and patients should perform anonymous authentication. The steps described further are carried out in the process of the patient’s authentication phase (Algorithm 1).

When the patient reaches the doctor’s spot, the MCU of the patient sends $\rho_{i}X$ to the MCU of the corresponding doctor (line 17 in Algorithm 1). Likewise, the MCU of the corresponding doctor sends $FID_{di}X$ to the patent’s MCU (line 18 in Algorithm 1). After this phase, the patient’s MCU computes $f=FID_{di}X.\;\rho_{i}$ (line 19 in Algorithm 1). Similarly, the doctor’s MCU computes $f=\rho_{i}X.FID_{di}$ (line 19 in Algorithm 1). Moreover, the patient’s MCU calculates $f_{1}=VID_{p_{i}}⨁H\left(f\right)$ and sends $f_{1}$ to the MCU of the doctor (line 20 in Algorithm 1). Once $f_{1}$ is received, the doctor’s MCU computes the validation ID of the patient’s as $VID_{p_{i}}=f_{1}⨁H\left(f\right)$. As a result, the computation time is drastically reduced due to the reduction in re-authentication time.

After computing the validation ID of the patient, the doctor’s MCU checks $e\left(VID_{p_{i}}X,VID_{d_{i}}\right)=Z$ in the blockchain network (line 21 in Algorithm 1). In this case, blockchain technology is used without the association of the TE. Only authenticated IoHT users can access this data since confidential information is stored in the blockchain. Attempts to hack the block by an intruder will have an impact on the subsequent blocks affecting the entire blockchain network.

**Proof of validation.**  $e\left(VID_{p_{i}}X,VID_{d_{i}}\right)=e\left(\rho_{i}\left(a+b\right)X,\left(\frac{1}{a+b}\right)X\right)\;=e{\left(X,X\right)}^{\rho_{i}\left(a+b\right)/\left(a+b\right)}\;=e{\left(X,X\right)}^{\rho_{i}}\;=Z$ □

Finally, the MCU of the doctor picks the fake identity of the patient $FID_{p_{i}}$ from the blockchain network (line 22 in Algorithm 1), and creates the authentication acknowledgment as $AA=\left(FID_{p_{i}},\;FID_{d_{i}},H\left(\;FID_{p_{i}},\;FID_{d_{i}}\right)\right)$. This acknowledgment will be transmitted to all the doctor’s MCUs to avoid re-authentication of the patients. Moreover, doctor’s MCU also computes $f_{2}=VID_{p_{i}}⨁FID_{d_{i}}$ and this value of $f_{2}$ is given to the patient’s MCU. Thus, the patient authenticates the doctor anonymously by extracting the fake identity of the doctor as $FID_{d_{i}}=VID_{p_{i}}⨁f_{2}$ (line 23 in Algorithm 1). With this step, the patient’s anonymous authentication process ends and the patient’s MCU is ready for data transfer. 

### 4.7. Doctor’s Anonymous Authentication 

The doctor provides confidential information such as medical prescriptions, diagnosis data, etc., to the patient in a secure way. Hence, it is necessary for a patient to authenticate the doctor before receiving confidential information from the doctor. Trust between the patient and doctor is mandatory to receive confidential information. The process of the doctor’s anonymous authentication is presented in Algorithm 2. In this procedure, the doctor’s MCU receives the following parameters $\left(VID_{di},FID_{di},c_{i},x,b,y_{3},y_{5},y_{6},y_{7}\right)$ from the TN (line 1 in Algorithm 2). Based on these values, the doctor’s MCU computes $l_{1}=FID_{d_{i}}⨁y_{7}$ (line 2 in Algorithm 2) and $l_{2}=VID_{p_{i}}⨁y_{6}$ (line 2 in Algorithm 2). Finally, the values of $l_{1},\;l_{2}$ and $c_{i}$ are sent to the patient’s MCU (line 4 in Algorithm 2). Once, these values are received, the patient’s MCU recovers $y_{6},\;y_{7}$ and checks $y_{6}X=\left(y_{1}+y_{6}\beta \right)$ as indicated in lines 5–7 in Algorithm 2. If this condition gratifies, the patient accepts the doctor’s confidential information. The values of $y_{7}$ and $y_{6}$ are recovered as $y_{7}=FID_{d_{i}}⨁l_{1}$ and $y_{6}=VID_{p_{i}}⨁l_{2}$. After the finalization of this phase, the doctor’s MCU is authenticated and transferring biotic information from the patient to the doctor can be performed.

**Proof of validation.**  $y_{7}X=\left(c_{i}+y_{6}b\right)X\;=\left(c_{i}X+y_{6}bX\right)\;=y_{1}+y_{6}\beta$ □

### 4.8. Transfer of Biotic Information from the Patient to the Doctor

In the next phase of the proposed authentication algorithm, the transfer of biotic information from the patient to the doctor starts. To send the confidential biotic information (ω) of the patient to another doctor in the network, the MCU of the patient chooses two random numbers $t_{1},\;t_{2}$ $\in Z_{q}^{*}$ (line 8 in Algorithm 2) and calculates the following parameters $u_{1},v_{1}$, $m_{1}\;and\;m_{2},$ as indicated in lines 9–12 in Algorithm 2 using the public parameters of the patient and doctor.

Once the parameters are calculated, the cipher test $\left(u_{1},v_{1}\right)$, $m_{1}$ and $m_{2}$ values are sent to the doctor (line 13 in Algorithm 2). The doctor checks the condition $e\left(u_{1},Sk_{d_{i}}.\;m_{1}+m_{2}\right)=g^{t_{1}}$ based on his secret key (line 14 in Algorithm 2). If the condition gratifies, then the confidential biological information (ω) of the patient is accepted, or it is discarded if the condition is not satisfied.

**Proof of validation.**  $e\left(u_{1},Sk_{d_{i}}.\;m_{1}+m_{2}\right)=e\left(t_{1}\left(Pk_{p_{i}}+H\left(\omega \right)X\right),Sk_{d_{i}}.\;m_{1}+m_{2}\right)\;=e\left(t_{1}\left(Pk_{p_{i}}+H\left(\omega \right)X\right),Sk_{d_{i}}.\;t_{2}X+{\left[Sk_{p_{i}}+H\left(\omega \right)\right]}^{-1}X-t_{2}Pk_{d_{i}}\right)\;=e\left(t_{1}\left(Pk_{p_{i}}+H\left(\omega \right)X\right),Sk_{d_{i}}.\;t_{2}X+{\left[Sk_{p_{i}}+H\left(\omega \right)\right]}^{-1}X-t_{2}Sk_{d_{i}}X\right)\;=e\left(t_{1}\left(Sk_{p_{i}}X+H\left(\omega \right)X\right),{\left[Sk_{p_{i}}+H\left(\omega \right)\right]}^{-1}X\right)\;=e\left(t_{1}\left(Sk_{p_{i}}+H\left(\omega \right)\right)X,{\left[Sk_{p_{i}}+H\left(\omega \right)\right]}^{-1}X\right)\;=e{\left(X,X\right)}^{\frac{t_{1}\left(Sk_{p_{i}}+H\left(\omega \right)\right)}{Sk_{p_{i}}+H\left(\omega \right)}}\;=e{\left(X,X\right)}^{t_{1}}\;=g^{t_{1}}$ □

### 4.9. Transfer of Medical Prescription from the Doctor to Patient 

The next phase of algorithm execution, based on the received biotic information of the patient, starts the process of transfer of medical prescription from the doctor to the patient. To send confidential information to the patient such as medical prescriptions ($\omega^{\prime }$) prepared by the doctor, the MCU of the doctor chooses two random numbers $t_{3},t_{4}$ $\in Z_{q}^{*}$ (line 15 in Algorithm 2) and calculates the $u_{2},\;v_{2}$, $m_{3}$ and $m_{4}$ parameters using the public parameters of the patient and doctor according to relations presented in lines 16–19 in Algorithm 2.

Once the parameters are calculated, the cipher test $\left(u_{2},v_{2}\right)$, $m_{3}$ and $m_{4}$ values are sent to the patient (line 20 in Algorithm 2). The patient checks the condition $e\left(u_{2},Sk_{p_{i}}\;m_{3}+m_{4}\right)=g^{t_{3}}$ based on his secret key (line 21 in Algorithm 2). If the condition gratifies, then the confidential medical prescription ($\omega^{\prime }$) sent by the doctor to the patient is accepted or else discarded. If the patient moves from one location to another location without the involvement of a TE, the new doctor takes the data of the patient from the blockchain. As a result, there is no re-authentication of the patient, and this can contribute to a reduction in the authentication time.

**Proof of validation.**  $e\left(u_{2},Sk_{p_{i}}\;m_{3}+m_{4}\right)=e\left(t_{3}\left(Pk_{d_{i}}+H\left(\omega^{\prime }\right)X\right),Sk_{p_{i}}\;m_{3}+m_{4}\right)\;=e\left(t_{3}\left(Pk_{d_{i}}+H\left(\omega^{\prime }\right)X\right),Sk_{p_{i}}\;t_{4}X+{\left[Sk_{d_{i}}+H\left(\omega^{\prime }\right)\right]}^{-1}X-t_{4}Pk_{p_{i}}\right)\;=e\left(t_{3}\left(Sk_{d_{i}}X+H\left(\omega^{\prime }\right)X\right),Sk_{p_{i}}\;t_{4}X+{\left[Sk_{d_{i}}+H\left(\omega^{\prime }\right)\right]}^{-1}X-t_{4}Sk_{p_{i}}X\right)\;=e\left(t_{3}\left(Sk_{d_{i}}+H\left(\omega^{\prime }\right)\right)X),{\left[Sk_{d_{i}}+H\left(\omega^{\prime }\right)\right]}^{-1}X\right)\;=e\left(t_{3}\left(Sk_{d_{i}}+H\left(\omega^{\prime }\right))X\right),{\left[Sk_{d_{i}}+H\left(\omega^{\prime }\right)\right]}^{-1}X\right)\;=e\left(t_{3}\left(Sk_{d_{i}}+H\left(\omega^{\prime }\right))X\right),{\left[Sk_{d_{i}}+H\left(\omega^{\prime }\right)\right]}^{-1}X\right)\;=e{\left(X,X\right)}^{\frac{t_{3}\left(Sk_{d_{i}}+H\left(\omega^{\prime }\right)\right)}{Sk_{d_{i}}+H\left(\omega^{\prime }\right)}}\;=e{\left(X,X\right)}^{t_{3}}\;=g^{t_{3}}$ □

### 4.10. Handover Mechanism and Integrity Preservation

In many real-life cases, the patient needs to receive medical opinions from different doctors, or specialists from different medical fields need to exchange patient medical information among them. The proposed authentication algorithm ensures authentication even for such information exchange. For instance, if the current doctor wants to share/send the confidential information of the patient (ω) to another doctor, the current doctor chooses three random numbers $d_{1},\;d_{2},d_{3}\in Z_{q}^{*}$ (line 22 in Algorithm 2). The algorithm in the next phase computes the following values $D_{1}$, $D_{2}$, $D_{3}$, $D_{4}$ and $D_{5}$ according to relations in line 23 in Algorithm 2, and the value of the parameter $Q_{i}$ according to the relation in line 24 in Algorithm 2.

Then, the current doctor sets $\sigma =\left(D_{4},\omega \right)$ as the signature of a confidential biological message (line 25 in Algorithm 2). Because of the unique nature of the signature that is attached to the confidential biological message, the message’s integrity will be preserved. The integrity of the signature will be preserved since it cannot be modified or altered by anyone. Then, the current doctor’s MCU sends $\left(Q_{i},TS,\omega ,\sigma ,D_{3},FID_{pi}\right)$ to another doctor’s MCU in the network (line 26 in Algorithm 2). Here, the TS signifies the time stamp at which the confidential message is created. Once the confidential message is received, the new doctor’s MCU in the network calculates $\partial_{i}=H\left(\omega \times D_{4}\right)$ from signature and checks the condition $Q_{i}\alpha =\partial_{i}D_{5}$ (line 27 in Algorithm 2). If it gratifies the condition, a confidential message (ω) is accepted by the MCU of a new doctor or it is rejected if the condition is not satisfied.

**Proof of validation.**  $Q_{i}\alpha =\partial_{i}\left(\;d_{1}+d_{2}+d_{3}\right)\alpha \;=\partial_{i}\left(\;d_{1}\alpha +d_{2}\alpha +d_{3}\alpha \right)\;=\partial_{i}\left(\;D_{1}+D_{2}+D_{4}\right)\;=\partial_{i}\left(\;D_{3}+D_{4}\right)\;=\partial_{i}D_{5}$ □

### 4.11. Revocation

Even when the authentication between users is successful, there may be a possibility that the doctors in the network may send fake information to the next doctor. In this paper, such activity is assumed as malicious misbehavior of medical staff. In that situation, the TE revokes the current misbehaving doctor from the network and marks his identity in the block list. Thus, further transmissions cannot be performed by the misbehaved doctor. For instance, let us assume that a fake message $\omega^{*}$ is sent by the misbehaved doctor to the other doctor in the network, i.e., the authentication parameters sent are $\left(TS,\omega^{*},\sigma ,FID_{di}\right)$. Once these parameters are received, knowing that the message is a fake message, the new doctor sends these parameters to the TE. Upon receiving $\left(TS,\omega^{*},\sigma ,FID_{di}\right)$, the misbehaved doctor with a fake identity will be removed.

Moreover, the TE sends a combination $\left(FID_{di},H\left(FID_{di},b\right)\right)$ to all the doctors in the network. Upon receiving this, the doctor’s MCU computes the parameter $ss=H\left(FID_{di},b\right).$ If the parameter ss is equal to the received $H\left(FID_{di},b\right)$, then the $FID_{di}$ will be stored in the block list. Hence, the doctor with the fake identity $FID_{di}$_,_ will not be allowed to proceed further in the IoHT network.

## 5. Security Analysis

This section deals with the defense of the proposed authentication framework against various types of attacks. The defense mechanism of the proposed framework against different assaults is described as follows.

### 5.1. Impersonation Attack

In impersonation attacks, the intruder pretends to be an authorized user to perform the impersonation attack. In the IoHT network, an external attacker must find the secret parameters of the authenticated entities to carry out an impersonation attack by pretending to be an authorized user. The random numbers such as $\rho_{i},k\in Z_{q}^{*}$ are chosen by the TE and are secretly provided to the patient in an offline manner. Similarly, the random values $c_{i},x\in Z_{q}^{*}$ for the doctors are secretly chosen by the TE and are provided to them. Hence, it is difficult for an intruder to calculate these random numbers due to the fact that such calculation belongs to the discrete log problem. Moreover, the secret parameters such as $\rho_{i}$ and $FID_{di}$ are provided secretly to the patient and doctor by the TE in an offline manner. These values are transferred between the entities during the anonymous authentication process. Due to all these reasons, it is hard for an intruder to pretend as a real entity and trace the values. Hence, the suggested protocol can withstand impersonation attacks.

### 5.2. Bogus Message Attack

To perform a bogus/fake message attack, the intruder wants to create a new fake message which is similar to the original message. However, in the authentication scheme proposed in this work, each message is attached with a signature. When the current doctor is transferring the confidential biological data of the patient to another doctor in the IoHT network, i.e., during handover authentication, the current doctor sets the signature $\sigma =\left(D_{4},\omega \right)$ and sends it to the next doctor (line 25 in Algorithm 2). In this case, the value of $D_{4}$ is calculated based on the value of $d_{3}\in Z_{q}^{*}$, which is a random number. 

Moreover, the computation of $D_{1},D_{2}$ and $Q_{i}$ involves the random numbers $d_{1},\;d_{2},d_{3}\in Z_{q}^{*}$ (lines 22 and 24 in Algorithm 2). As the numbers are random in nature, it is difficult for an attacker to trace the signature and the confidential message integrity is preserved. Moreover, during the transfer of biological information of the patient to the doctor, the biological information is secured using the private key of the patient $(Sk_{p_{i}}$). Only the authenticated doctor in the network with his secret private key ($Sk_{d_{i}}$) can obtain the confidential data of the patient. Similarly, during the transfer of a medical prescription from the doctor to the patient, the information is securely transferred via the secret key of the doctor ($Sk_{d_{i}}$). In this case, only the authenticated patient with its secret key ($Sk_{p_{i}}$) can read the medical prescription. Hence, the proposed algorithm offers a defense against fake message attacks. 

### 5.3. Message Modification Attack

To perform a message modification attack, the intruder should modify the content of the message within the stipulated time and send the modified message to the authenticated users in the network. However, in the proposed authentication scheme, the current doctor’s MCU sends $\left(Q_{i},TS,\omega ,\sigma ,D_{3},FID_{pi}\right)$ to another doctor’s MCU in the network (line 26 in Algorithm 2). The current doctor sets $\sigma =\left(D_{4},\omega \right)$ as the signature of a confidential biological message (line 25 in Algorithm 2). Because of the unique nature of the signature that is attached to the confidential biological message, the message’s integrity will be preserved. Here, the value of $D_{4}$ is computed as $d_{3}\alpha$ which involves random value $d_{3}\in Z_{q}^{*}$ (line 23 in Algorithm 2). However, the random value is known only to the current doctor and it lasts for a short duration. The random value changes during each subsequent transfer of information between the doctors. Even though, if an intruder cracks this random value, it is still difficult to trace the subsequent transfer of data. Hence, the suggested authentication scheme is resistant to message-modification attacks. 

### 5.4. Revocation Atack

The revocation attack is the mechanism by which the unauthenticated entity is removed from the network. In the case of a developed authentication algorithm, the end users, both the patient and doctor, are anonymously authenticated by using their fake/dummy identity. However, there may be a situation when the current doctor may be compromised and send fake information about the patient to the subsequent or other doctors in the network. In this case, the identity of the current doctor should be revealed, and his identity should be kept on the blocklist and revoked from the network. For instance, a fake message $\omega^{*}$ is sent by the misbehaved doctor to the other doctor in the network, i.e., $\left(TS,\omega^{*},\sigma ,FID_{di}\right)$. Once these parameters are received, knowing that this message is a fake message, the new doctor sends these parameters to the TE. Once these parameters are received, the misbehaved doctor with the fake identity ($FID_{di}$) will be removed. Moreover, the TE publishes this fake ID and places it in the blocklist to avoid further transfer of information by this misbehaved doctor in the network. Thus, the other authenticated doctors in the network will avoid further communication with the current misbehaved doctor. 

### 5.5. Non-Repudiation Attack

The non-repudiate attack is the concept of an attack in which the end users deny the acceptance of the received information. However, in the case of the authentication scheme proposed in this work, only after the successful authentication of the patient and doctor by the TE are the authenticated entities (patient/doctor) allowed to participate in the IoHT network communication. Therefore, the end users cannot repudiate after transferring the related data. Either during the transfer of biotic information of the patient to the doctor by the patient’s device or during the transfer of medical prescription prescribed by the doctor to the patient, secret keys of the corresponding entities are used to hide the information. Hence, either the doctor or the patient cannot repudiate data after sending it. 

### 5.6. Anonymity and Privacy-Preservation Attack

The proposed work uses fake identities and signatures provided by the TE for transferring confidential information between the end users. This type of security threat is a sort of man-in-the-middle attack. To communicate with entities in the IoHT, a fake identity is used by the end user. This fake identity is exposed to other entities during data transfer. Moreover, in the TN, dummy/fake identities are mapped to the true identities. Therefore, even if the fake identities are captured, it will not provide any information about the true user identities. Thus, the authorized user can anonymously authenticate the specific user and maintain privacy. 

### 5.7. Unlinkability Attack

A lack of connectivity between the two simultaneous messages that are transferred between the end users is referred to as an unlinkability attack. The suggested scheme achieves unlinkability by the usage of short-time secret key generation during the transfer of information. During the transfer of confidential biological information (ω) of the patient to another doctor in the network, the MCU of the patient chooses two random values $t_{1},t_{2}$ $\in Z_{q}^{*}$ (line 8 in Algorithm 2) and calculates the cipher text $\left(u_{1},v_{1}\right)$, $m_{1}$,$m_{2}$ and send it to the doctor (lines 9–13 in Algorithm 2). Here, the computation of $m_{2}$ involves the usage of a secret key ($Sk_{d_{i}}$) whose validity is for a short duration. Moreover, the values of $\left(u_{1},v_{1}\right)$, $m_{1}$ involves $t_{1},t_{2}$ which are the random values generated only during the transfer of data at a specific time interval. Once, the transfer process is completed, the values need to be changed for further communication. Similarly, during the transfer of confidential information such as medical prescriptions ($\omega^{\prime }$) prepared by the doctor to the patient, the MCU of the doctor chooses two random values $t_{3},t_{4}$ $\in Z_{q}^{*}$ and calculates the cipher test $\left(u_{2},v_{2}\right)$, $m_{3}$ and $m_{4}$ (lines 16–20 in Algorithm 2). As these random values are periodically changed, there is complete unlinkability in the suggested authentication framework. 

### 5.8. Sybil Attack

In a Sybil attack, one or more fake identity patients (intruders) may send spurious information regarding their biological data at the same time to the authenticated doctor in the network. As a result, the authenticated doctor becomes busy in receiving this fake information. Moreover, the doctor will not be able to serve the authenticated patient, since it becomes extremely busy. In the case of the proposed authentication scheme, to send fake information by the patient’s device to the doctor, the attacker (fake patient(s)) should crack the values of $\rho_{i}$ to compute f and $f_{1}$ (lines 19–20 in Algorithm 1). However, the value of $\rho_{i}$ is provided to the patient in an offline way during the initial registration process by TE (line 9 in Algorithm 1). Moreover, the computation of $f_{1}$ involves $VID_{p_{i}}$ (line 20 in Algorithm 1), where $f_{1}=VID_{p_{i}}⨁H\left(f\right).$ Here, $VID_{p_{i}}$ is also provided to the authenticated patient by the TE. Thus, manipulating these values and sending multiple fake requests to the authenticated doctor in the IoHT is not possible and the proposed authentication scheme offers protection against Sybil’s attack.

### 5.9. Replay Attack

In the reply attack, the information is captured during the transmission and transmitted after a certain interval of time by an external attacker. To avoid this attack, in the proposed authentication scheme a timestamp is attached during the transfer of information. More specifically, during the handover authentication phase, the current doctor’s MCU sends $\left(Q_{i},TS,\omega ,\sigma ,D_{3},FID_{pi}\right)$ to another doctor’s MCU in the network. 

Here, the TS signifies the time stamp at which the confidential information is generated. Once the confidential information is received, the new doctor’s MCU in the network verifies whether $\left|t_{j}-t_{i}\right|<△t$, where △t is the time delay between internal end users. If the time delay is unreasonable, the information is simply rejected by the new doctor’s MCU. As a result, the proposed authentication method can withstand the Replay attacks.

## 6. Performance Analysis

The performance of the proposed authentication algorithm for the described IoHT network is analyzed in terms of computational, communication and storage costs. The brief discussion regarding each analysis is explained as follows. 

### 6.1. Computational Overhead

Computational overhead refers to the time required to complete the cryptographic operations dedicated to the authentication of the IoHT users (i.e., patients and doctors). In this work, random computations are performed for 100 simulations and the mean time of all computations is calculated as computational overhead. The performance of the proposed authentication scheme is compared with similar state-of-the-art schemes such as those published by Kumar et al. [44], Liu et al. [49], Jegadeesan et al. [50], Debiao et al. [51] and Jia et al. [52]. The simulations are performed on the server with the next hardware characteristics (Table 1): the processor Core i7 with 16 GB RAM and the 2.20 GHz CPU frequency having the 64-bit operating system with Cygwin software containing the Pairing-Based Cryptography (PBC) library [53]. Cryptographic operations including the one point cryptographic multiplication $\left(T_{m}\right)$, the one point addition $\left(T_{a}\right)$, the exponential operation $\left(T_{e}\right)$, the pairing operation $\left(T_{p}\right)$, the hashing function $\left(T_{h}\right)$, and the exclusive OR operation $\left(T_{xor}\right)$ are involved in the calculations. The time duration for each of these calculations performed on the server with specified hardware characteristics is shown in Table 1. 

Table 2 shows the comparison of the relations for the calculation of the computation time for analyzed authentication schemes. The relations enable the calculation of the authentication time costs required for the above-mentioned schemes for the comparison with the authentication time costs of the authentication scheme proposed in this work. The value of n in relations presented in Table 2 defines the number of users (public keys) participating in the authentication process. 

**Figure 2:**
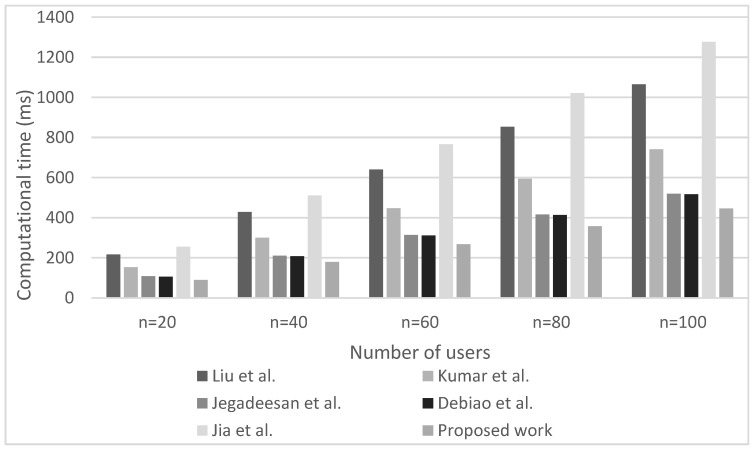
Computation overhead of analyzed authentication schemes at the patient’s side (Liu at al. [49], Kumar at al. [44], Jegadeesan et al. [50], Debiao et al. [51] and Jia at al. [52]).

**Figure 3:**
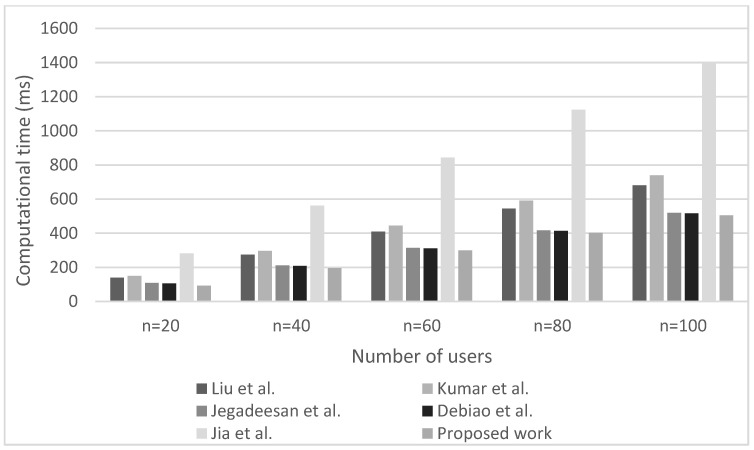
Computation overhead of analyzed authentication schemes at the doctor’s side (Liu at al. [49], Kumar at al. [44], Jegadeesan et al. [50], Debiao et al. [51] and Jia at al. [52]).

Additionally, Figure 2 and Figure 3 present the computational overhead in terms of computation time for executing the authentication process in the case of various authentication schemes at the patient and doctor sides, respectively. The simulations are performed ranging from 20 to 100 simultaneously authenticated users. The figures clearly indicate that as the number of IoHT users increases, the computation overhead for authenticating them also increases. Concerning the relations presented in Table 2, this is the expected result since the time for performing the authentication process is directly proportional to the number n of users participating in the authentication process.

Moreover, the results presented in Figure 2 and Figure 3 indicate that for any number of simultaneously authenticated users, the computation time for performing the authentication process is lowest for the proposed authentication scheme. This has been achieved since the proposed authentication scheme, in contrast to other analyzed state-of-the-art authentication schemes (Table 2), involve only one hashing operation, two-point multiplication operations, and four XOR operations at the patient side for authentication purposes. For example, when compared to the other authentication schemes, the proposed authentication scheme lasts only 4.4583 ms for performing the authentication process of a single user, whereas in the case of the scheme proposed by Liu et al. in [49] is 10.65 ms, by Kumar et al. in [44] is 7.41 ms, by Jegadeesan et al. in [50] is 5.18 ms, by Debiao et al. in [51] is 5.16 ms and by Jia et al. in [52] is 12.76 ms for the authentication of the single patient. This authentication period is the lowest when compared to other analyzed state-of-the-art authentication schemes. According to Figure 3, similar results have been obtained for the computation time of analyzed authentication schemes on the doctor’s side. Therefore, the results presented in Figure 2 and Figure 3 confirm that the proposed authentication scheme outperforms other state-of-the-art authentication schemes in terms of the computation time needed for performing the authentication process.

### 6.2. Communication Overhead

**Figure 4:**
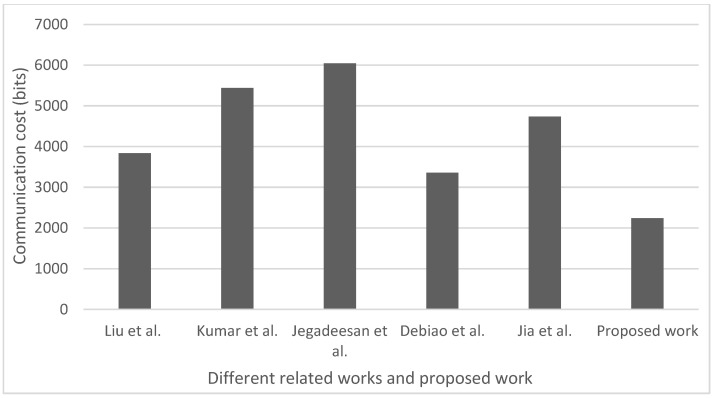
Communication overhead for analyzed authentication schemes (Liu at al. [49], Kumar at al. [44], Jegadeesan et al. [50], Debiao et al. [51] and Jia at al. [52]).

The number of bits exchanged during the transformation of information in the frame of the authentication process between the IoHT users is referred to as communication overhead. Figure 4 shows the comparison of the communication overhead of the proposed authentication scheme and similar state-of-the-art authentication schemes such as those presented by Liu et al. in [49], Kumar et al. in [44], Jegadeesan et al. in [50], Debiao et al. in [51] and Jia et al. in [52]. 

Figure 4 shows that the suggested authentication scheme outperforms the authentication schemes proposed in the stated related works in terms of communication overhead. This is a consequence of the low number of bits used in the proposed authentication scheme for presenting the cipher test values. More specifically, during the transfer of the patient’s biological data from the patient to the doctors, the cipher test values ($\left(u_{1},v_{1}\right)$,$\;m_{1}$ and $m_{2}$) are sent to the doctor (lane 13 in Algorithm 2). Similarly, when the medical prescription is transferred from the doctor to the patient, the doctor sends the cipher test values ($\left(u_{2},v_{2}\right)$,$\;m_{3}$ and $m_{4}$) to the patient (lane 20 in Algorithm 2). The values of $u_{1}$,$\;u_{2}$ involve the hashing output and the public key of the patient and doctor which are computed in 160 bits. The value of $v_{1}$ and $v_{2}$ are the output of the hash function which are also computed in 160 bits. The values of $m_{1},m_{2},m_{3}$ and $m_{4}$ involves the hash output and the public and secret keys of the doctor and patient, respectively. 

Therefore, the entire communication overhead of the proposed authentication scheme is computed and transferred in 2240 bits (Figure 4). When compared to the authentication costs of other authentication schemes presented in Figure 4, the proposed authentication scheme proposed by Liu et al. in [49] needs 3840 bits, by Kumar et al. in [44] requests 5440 bits, by Jegadeesan et al. in [50] needs 6048 bits, by Debiao et al. in [51] needs 3348 bits and by Jia et al. in [52] needs 4736 bits for the authentication of a single patient. Thus, the proposed authentication scheme results in significantly lower communication overhead. 

The communication overhead of the proposed authentication scheme is 33,33% lower than the communication overhead of the authentication scheme having the second lowest communication overhead. This confirms the superiority of the proposed authentication scheme in terms of communication overhead when compared with other prominent authentication schemes. 

### 6.3. Storage Cost

Storage overhead plays a key role in the performance evaluation of the authentication process. Storage costs account for the number of bits that must be stored in the memory of user devices during the authentication process. Table 3 shows the results obtained for the storage cost of analyzed authentication schemes. The number of bits stored in the patient’s MCU and the doctor’s MCU should be as small as possible. 

In the proposed authentication scheme, the patients are required to store parameters $\rho_{i},k,FID_{p_{i}}\in Z_{q}^{*}$. Similarly, doctors are required to store the parameters $c_{i},x,FID_{di}\in Z_{q}^{*}$. To have accomplished authentification, only these parameters are sufficient to be stored by the patient and doctor MCU in the case of the proposed authentication scheme. Since these parameters are used for the key generation process, storing these values is essential for the efficient transfer of information between the doctor and patient. Thus, the storage overhead at the patient’s and doctor’s sides according to Table 3 is 480 bits. As a result, the overall storage cost of the proposed authentication scheme is 960 bits. For instance, the proposed authentication scheme requires only 480 bits to be stored on the patient’s side for verification (Table 3), whereas the authentication scheme proposed by Kumar et al. in [44] requests 2176 bits, by Jegadeesan et al. in [50] needs 1792 bits, by Debiao et al. in [51] needs1088 bits and by Jia et al. in [52] needs 1184 bits for the authentication of the single patient.

These results present a significant improvement when compared with the storage costs of existing schemes (Table 3). Therefore, the proposed authentication scheme improves the authentication process through the exploitation of a significantly lower amount of patient and doctor MCU memory. In comparison with other relevant authentication schemes, a notable decrease in the number of bits that must be saved during the authentication process gives a significant implementation advantage to the proposed authentication scheme.

## 7. Conclusions

In this manuscript, an efficient certificateless blockchain-based anonymous privacy-preserving authentication scheme is proposed. This work is mainly focused on the reliable and efficient transfer of authentication information between the doctor and patient user device in the IoHT environment. A detailed explanation of the algorithm for performing authentication in the IoHT network is presented. The authentication algorithm is based on the generation of private keys which are used in the authentication process during cipher text validation. In addition, these keys are generated based on the lightweight elliptic curve method. Blockchain technology is used as an approach for achieving efficient authentication of the patient without the involvement of a trusted entity. An efficient authentication handover mechanism is also developed in the frame of the proposed authentication scheme and this mechanism enables the transfer of the patient’s data between the doctors in a secure way. Additionally, an efficient revoking mechanism is suggested to remove the potential misbehaving doctors from the IoHT network. The obtained results for the performance analyses of the proposed authentication scheme prove that the proposed authentication algorithm can withstand different possible security threats. Moreover, a performance comparison with other related state-of-the-art authentication schemes shows that the proposed authentication scheme enables significant improvements in terms of computation, communication and storage overhead.

The main limitation of the proposed authentication scheme is the dynamic increase in the patient’s and doctor’s data stored in the trusted authority. Since the bulk of data is stored in a trusted authority, data accessibility can become challenging when the amount of data significantly increases. However, if the fog computing concept is incorporated, patients’ data can be temporarily stored closer to the authenticated doctor for frequent and faster data access. Therefore, performance analyses of this concept based on fog computing will be the main focus of future research.

Moreover, the algorithm proposed in this paper can be used in the practical implementations of an efficient mobile control unit for both, patients and doctors. As a result, the computational operations are performed in a faster way, which reduces the transmission delay of the confidential data. Thus, the speed of the authentication process at the devices in the IoHT network can be increased. Moreover, only a minimum number of bits need to be stored in the memory of end devices, which reduces the memory demand and leads to the reduced power consumption of mobile devices.

In addition, the location privacy of wireless body area network users will be one of the possible future extensions of this research work. Location privacy and security should be preserved while accessing the wireless body area network from various locations during the user’s movement. Further, an automatic billing scheme for the medical prescriptions provided by the doctor for accessing the patient data can be incorporated into IoHT networks and this is also a research topic of interest. Finally, future work can be extended in different areas of applications such as education, supply chain management, vehicle ad-hoc networks and even government organizations. 

## Figures and Tables

**Table 1:** Time duration for cryptographic operations.

Hardware Characteristics of the Simulation Server	Cryptographic Operation	Time Duration in Milliseconds (ms)
Processor: Core i7 RAM: 16 GB Frequency: 2.20 GHz Operating system: 64-bit Software: Cygwin with PBC library	One point multiplication $\left(T_{m}\right)$	2.226
	One point addition $\left(T_{a}\right)$	0.001
	Exponential operation $\left(T_{e}\right)$	3.85
	Pairing operation $\left(T_{p}\right)$	2.91
	Hashing function $\;(T_{h})$	0.0023
	Exclusive OR operation $\left(T_{xor}\right)$	0.001

**Table 2:** Relations for calculation of computational time for analyzed authentication schemes.

Authentication Schemes	Authentication Time at the Patient Side (ms)	Authentication Time at Doctor Side (ms)
Liu et al. [49]	$nT_{p}+\left(n+1\right)T_{h}+\left(2n+1\right)T_{e}$	$nT_{p}+\left(n+1\right)T_{e}+nT_{h}$
Kumar et al. [44]	$(n+1)T_{p}+\left(n+1\right)T_{h}+\left(2n+1\right)T_{m}$	$nT_{p}+\left(2n+1\right)T_{h}+\left(2n+1\right)T_{m}$
Jegadeesan et al. [50]	$(n+1)T_{p}+\left(n+1\right)T_{h}+\left(n+1\right)T_{m}$	$(n+1)T_{p}+nT_{h}+\left(n+1\right)T_{m}$
Debiao et al. [51]	$(n+1)T_{m}+\left(n+1\right)T_{h}+nT_{p}+nT_{a}$	$(n+1)T_{m}+\left(2n+1\right)T_{h}+nT_{p}$
Jia et al. [52]	$4nT_{m}+nT_{e}+5nT_{h}$	$nT_{p}+5nT_{m}+\left(2n+1\right)T_{a}+5nT_{h}$
Proposed work (authentication scheme)	$2nT_{m}+nT_{h}+4nT_{xor}$	$nT_{m}+3nT_{xor}+nT_{h}+nT_{p}+nT_{a}$

**Table 3:** Storage overhead for analyzed authentication schemes.

Authentication Schemes	Patient’s Side (Bits)	Doctor’s Side (Bits)
Kumar et al. [44]	2176	2176
Jegadeesan et al. [50]	1792	1792
Debiao et al. [51]	1088	160
Jia et al. [52]	1184	1024
Proposed work	480	480

## Nomenclature

**Notations**	**Explanation**
TE	Trusted entity
X	Point on the elliptic curve
q	Largest prime value
a,b	Random numbers of TE
α	Public parameter of TE
β	Authentication parameter of TE
*H*: {0, 1}*	Hash function
$p_{i}$, di	Patient and Doctor
$\rho_{i},k$	Random numbers for patient chosen by TE
$VID_{p_{i}}$	Validation ID for the patient
$FID_{p_{i}}$	Fake ID for the patient
$c_{i},x$	Random numbers for doctor chosen by TE
$VID_{di}$	Validation ID for doctor
$FID_{di}$	Fake ID for doctor
$Sk_{p_{i}}$	Secret key for patient
$Pk_{p_{i}}$	Public key for patient
$Sk_{d_{i}}$	Secret key for doctor
$Pk_{d_{i}}$	Public key for doctor
ω	Confidential biological information of $p_{i}$
$t_{1},t_{2}$	Random numbers chosen by the patient
$\left(u_{1},v_{1}\right)\&\;\left(u_{2},v_{2}\right)$	Cipher texts
$\omega^{\prime }$	Medical prescription of the doctor
$t_{3},t_{4}$,$\;d_{1},\;d_{2},d_{3}$	Random numbers chosen by the doctor
σ	Signature of a confidential biological message
TS	Timestamp
$\omega^{*}$	Fake message
⊕	Exclusive OR operation
